# Evaluation of a Virtual Reality Platform to Train Stress Management Skills for a Defense Workforce: Multisite, Mixed Methods Feasibility Study

**DOI:** 10.2196/46368

**Published:** 2023-11-06

**Authors:** Murielle G Kluge, Steven Maltby, Caroline Kuhne, Nicole Walker, Neanne Bennett, Eugene Aidman, Eugene Nalivaiko, Frederick Rohan Walker

**Affiliations:** 1 Centre for Advanced Training Systems Faculty of Health, Medicine & Wellbeing The University of Newcastle Callaghan Australia; 2 School of Biomedical Sciences & Pharmacy Faculty of Health, Medicine & Wellbeing The University of Newcastle Callaghan Australia; 3 School of Psychological Sciences College of Engineering, Science and Environment The University of Newcastle Callaghan Australia; 4 Army School of Health Australian Defence Force Canberra Australia; 5 Joint Health Command Department of Defence Canberra Australia; 6 Human and Decision Sciences Division Defence Science & Technology Group Edinburgh Australia

**Keywords:** virtual reality, workplace training, stress management, defense

## Abstract

**Background:**

Psychological stress-related injuries within first-responder organizations have created a need for the implementation of effective stress management training. Most stress management training solutions have limitations associated with scaled adoption within the workforce. For instance, those that are effective in civilian populations often do not align with the human performance culture embedded within first-responder organizations. Programs involving expert-led instructions that are high in quality are often expensive.

**Objective:**

This study sought to evaluate a tailored stress management training platform within the existing training schedule of the Australian Defense Force (ADF). The platform, known as Performance Edge (PE), is a novel virtual reality (VR) and biofeedback-enabled stress management skills training platform. Focusing on practical training of well-established skills and strategies, the platform was designed to take advantage of VR technology to generate an immersive and private training environment. This study aimed to assess the feasibility of delivering the VR platform within the existing group-based training context and intended training population. In this setting, the study further aimed to collect data on critical predictors of user acceptance and technology adoption in education, including perceived usability, usefulness, and engagement, while also assessing training impacts.

**Methods:**

This study used a mixed methods, multisite approach to collect observational, self-reported, and biometric data from both training staff and trainers within a real-world “on-base” training context in the ADF. Validated scales include the Presence Questionnaire and User Engagement Scale for perceived usefulness, usability, and engagement, as well as the State Mindfulness Scale and Relaxation Inventory, to gain insights into immediate training impacts for specific training modules. Additional surveys were specifically developed to assess implementation feedback, intention to use skills, and perceived training impact and value.

**Results:**

PE training was delivered to 189 ADF trainees over 372 training sessions. The platform was easy to use at an individual level and was feasible to deliver in a classroom setting. Trainee feedback consistently showed high levels of engagement and a sense of presence with the training content and environment. PE is overall perceived as an effective and useful training tool. Self-report and objective indices confirmed knowledge improvement, increased skill confidence, and increased competency after training. Specific training elements resulted in increased state mindfulness, increased physical relaxation, and reduced breathing rate. The ability to practice cognitive strategies in a diverse, private, and immersive training environment while in a group setting was highlighted as particularly valuable.

**Conclusions:**

This study found the VR-based platform (PE) to be a feasible stress management training solution for group-based training delivery in a defense population. Furthermore, the intended end users, both trainers and trainees, perceive the platform to be usable, useful, engaging, and effective for training, suggesting end-user acceptance and potential for technology adoption.

## Introduction

### Stress Management Training in the Workforce

The negative impacts of unmanaged stress exposure are well documented in first-responder populations [1,2]. Consequences of prolonged exposure to unmanaged psychological stress can include, but are not limited to, changes in cognition, judgment, motivation, and mood [3]. With prolonged exposure to stress, disruptions in mood and cognition can transition into diagnosable pathologies such as burnout, anxiety, depression, and trauma [4-6].

To protect their workforce and those under their care, many first-responder organizations have sought to deliver scalable and structured stress management training to their staff. Stress management training includes several different forms and components. A useful viewpoint is the definition of stress management training as “the application of any set of techniques (*e.g.*, exposure training, relaxation, biofeedback, and cognitive behavioural therapy) with the intent to improve the way people cope with stress” [7]. Furthermore, existing stress management training programs can be broadly classified into two types: (1) stress-inoculation training—repeated exposure to a stressor to develop tolerance (eg, outdoor adventure, live fire, and mission rehearsal) and (2) cognitive and psychological skills training conducted in a nonstressful setting. Stress inoculation training programs have been popular for military and first-responder training organizations when the stressor is predictable and likely (eg, physical and verbal altercation or combat [8,9]). Cognitive and psychological skills training, also often referred to as resilience training, can include mindfulness-based, cognitive-behavioral strategies and relaxation techniques [10-15]. Breathwork and mindfulness-based training interventions have documented efficacy in both clinical and nonclinical settings [16,17]. These strategies are being increasingly assessed in workforce and workplace contexts [18-21]. Cognitive strategies, including goal setting and emotional and attentional control, have also been shown to positively impact psychological well-being in military personnel [22-25]. Although there is growing evidence on the benefit of training cognitive stress management skills in first responders and similar occupational cohorts [26-30], further research is required to inform best practice strategies on how to effectively implement and scale training within and across organizations. A major challenge in this context is that many workplace and training organizations deliver training as scheduled activities and in groups.

The Australian Defence Force (ADF) has a well-established stress-management training platform (BattleSMART) based on cognitive behavioral therapy tailored to ADF members [31]. Consistent with other stress management programs, BattleSMART relies on the provision of instructional and theoretical materials on using cognitive and psychological skills [31]. Although well accepted, BattleSMART has been constrained, with respect to skill establishment, by the number of expert facilitators and the time required to deliver the program. Robustly establishing psychological skills, as is the case with other complex skills, requires extensive time for skill rehearsal and expert-led facilitation [18]. A major challenge connected to the delivery of practical stress management skills is that this type of training benefits from a private and focused training environment. Hence, it is typically facilitated in one-on-one sessions. However, like many training organizations, ADF typically organize and operate their activities via a group-based structure. A change from group-based to one-on-one training for stress management skills instruction would represent a significant burden to the organization.

### The Use of Virtual Reality for Stress Management Training

Virtual reality (VR) is an interesting solution for stress management training. VR provides a technical platform to place skill development and expert-led instruction “into the headset.” Moving from a human delivered to digitally delivered instruction can mitigate many issues associated with specialist workforce limitations and allow for flexibility in when and where training can occur. Although similar things can be said about many digital software solutions, the VR headset can create a private and immersive environment that can be particularly beneficial for both group-based delivery and the nature of the subject matter. Additional benefits of VR over other conventional 2D-based platforms include increased immersion, interaction through handheld controllers, a strong sense of presence, engagement, and student motivation [32-34]. Thus, VR-delivered training may provide a viable solution to circumvent the major challenges associated with group-delivered implementation of stress management training for large organizations, including defense.

Several VR and biofeedback-integrated training applications have already been trialed in military and police populations, including those targeting stress inoculation, passive relaxation, and breath control [9,35-38]. To our knowledge, however, there have been no comprehensive stress management training programs that teach a diverse range of stress management skills that are appropriate for first-responder organizations, such as the Australian military.

### The Performance Edge Stress Management Training Platform

To address the existing unmet needs for practical and scalable training of stress management skills for first-responder populations, we developed a new and comprehensive VR-based training platform called Performance Edge (PE) [39]. In collaboration with the ADF, PE was specifically developed to target an early career training population and be aligned with ADF values. Evaluation of the first PE module, which focused on training controlled breathing skills, demonstrated the in-principal suitability of the technology and training approach [39]. Building on the initial work, the modular PE platform was expanded to include 5 modules, each of which provided fundamental skill training for evidence-based stress management strategies adapted from cognitive-behavioral therapy and acceptance and commitment therapy. More details on the PE training concept, the framework and specific module content are provided in Multimedia Appendix 1.

This study has the following objectives:

1. Evaluate the feasibility and implementation of the 5-module PE prototype training package, delivered on-site at multiple military locations to its intended ADF target population, and within existing training activities and group-based, classroom settings.
2. Assess critical predictors of technology adoption (eg, perceived usability, and usefulness), engagement, and training impact.

## Methods

### PE Training Platform and Framework

The PE software was delivered on the Oculus Quest (Meta), a freestanding VR headset with 2 handheld controllers and an inside-out tracking system. A total of 20 headsets using the Oculus for Business Enterprise platform were used, which supported fleet management, content uploading, and battery monitoring. Respiratory signals were collected and integrated into VR training using a GoDirect respiratory belt (Vernier). A custom software application was developed and installed to translate and display the breathing traces and rates (breaths per minute) from the belt into the headset in real time to facilitate biofeedback for training.

The PE software application used in this study contained a full menu management system and 5 individual training modules. Each module focused on a specific skill or cognitive strategy adapted from cognitive-behavioral therapy and acceptance and commitment therapy principles (Table 1 and Multimedia Appendix 1). All PE modules included an introductory video, guided narration, personalized learning, practical training, user interactions, feedback, performance measures, and the opportunity for repetition.

**Figure 1:**
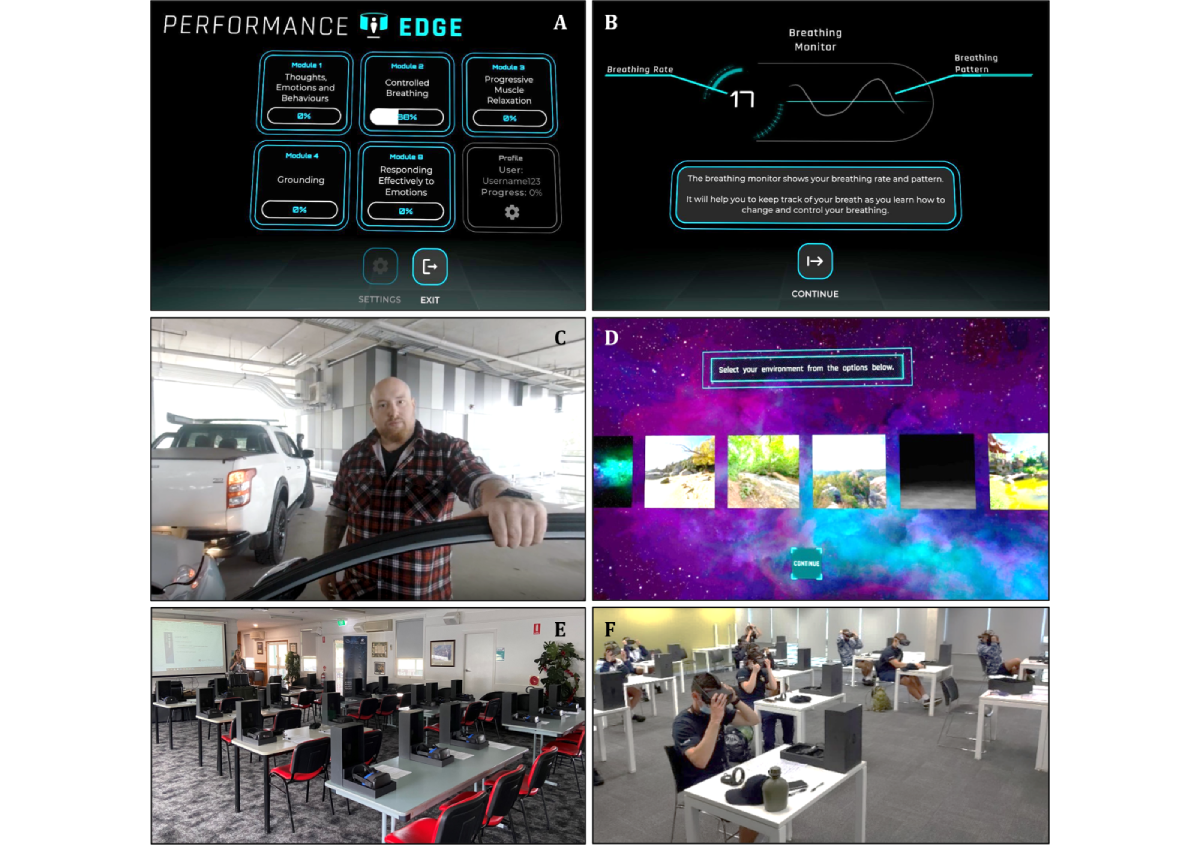
Performance Edge example screenshots and deployment: (A) main menu, (B) breathing monitor trace and breathing rate from module 2, (C) 360° video scene from module 1, (D) environment selection screen from module 3, (E) set-up in standard classroom, and (F) training under COVID-19 social distancing requirements.

The PE interface adopts a futuristic design that pays homage to space-themed computer games (Figures 1A-1D). Although the target audience was military, visual design features were created using a neutral pallet without specific reference to military design, situations, or terminology. This was in part due to military triservice-specific branding but also emphasized that the skills can be relevant for any stress-provoking situation, either work related or in everyday life. Alignment with ADF values was generated using a clear and directive tone for instructions, feedback, and explanations, with an emphasis on practical elements. All the exercises were designed to take advantage of the immersive nature and interactive capability of VR technology to create an engaging learning and training environment. Examples can be found in Multimedia Appendices 2 and 3, containing walk-through videos from the user's view within the VR headset.

**Table 1:** Performance Edge modules, features, and brief description of content.

Module	Module name	Description	Training and learning objective
M1	Thoughts, Emotions and Behaviors	Introduction to the training framework and concepts. This module contains 6 exercises including fun, pleasant, annoying, and confrontational elements with different computer-generated interface and 360-degree generated scenarios. In addition to guided narration, the module uses selection items for trainees to identify and distinguish emotions, thoughts, and physical reactions in response to the stimuli in the scene. See Multimedia Appendix 2.	Understand the connection and distinction between initial thoughts, emotions, and behaviors in response to challenging situations. Recognize and reflect on your thoughts and emotions and initial behavioral urges in response to different situations. Demonstrate that uncomfortable emotions and thoughts are sometimes reasonable, however acting on them is not always helpful. Demonstrate that different people respond differently to the same situation.
M2	Controlled Breathing	Practical training to develop controlled breathing skills. This module contains 7 escalating exercises with integrated live biofeedback (breathing rate and pattern). Live breathing traces and rates, presented as the breathing monitor, are used to facilitate knowledge growth, as a visual aid and guide to support skill development and to track performance and skill improvement throughout the training process. Difficulty increases through the training by removing visual aids and placing the trainee in distracting environments.	Understand the voluntary control of the breath as a tool to calm down the physiological body. Understand the impacts of physiological activity and psychological stress on the breath. Breath awareness. Skill development of controlling and maintaining a conscious, slow, and steady breathing rate.
M3	Progressive Muscle Relaxation	Guided exercises on progressive muscle relaxation. Trainees customize the experience, selecting level of instruction and guidance, visual aids, voice, and background environment. State mood and respiratory rates are collected at the start and after the exercise to visualize changes in mood and relaxation.	Understand the impact of stress on the physical body or musculature. Experience body awareness. Notice and reduce physical tension.
M4	Grounding	Eight practical exercises on how to use auditory, visual, and sensory cues to ground in the present moment with focus on regaining control over attention and awareness. Trainees are placed into different 360-degree environments with specifically designed, complex or targeted audio or visual imagery ranging from pleasant, thought provoking, annoying to confrontational in nature. Exercises combine guided narration to help focus attention on a specific stimulus and switch between stimuli. Interactive elements of a trigger pull, and selection items are used to support engagement and ensure participation in this cognitive skill. Advanced exercises instruct the trainee to use grounding after a challenging event and memory recall.	Provides a basic understanding of how challenging internal experiences can lead to being absorbed or overwhelmed. Practice progressively shifting attention toward different external cues. Understand the benefits of regulating attention in moments that are overwhelming or challenging. Practice using grounding to get out of your head and into the present moment and regain situational awareness.
M5	Responding Effectively to Emotions	This module includes 4 exercises on emotional awareness and accepting emotions in response to different situations and environments. Trainees are instructed to select emotions and their intensity from an expandable section element. The module contains guided narration on sensory awareness and acceptance exercises combined with active participation in increasingly challenging activities designed to elicit specific emotional responses. Computer-generated interface games include rigged outcomes, negatively valenced feedback, and social exclusion elements.	Understand that emotions are transient and the potential negative impact of avoidant strategies. Practice awareness of emotions Practice recognizing uncomfortable emotions, physical sensation, and automatic behavioral urges in response to (uncomfortable) emotions. Use contextualization to reduce the unhelpful impact of emotions. Practice using acceptance to response more effectively to (difficult or uncomfortable) emotions.

### Study Participants and Recruitment

Participants were recruited from the initial employment training (IET) programs at 3 military bases in Australia. The IET program is the first service-specific training that military personnel undergo after basic training. As both the existing stress management training program (BattleSMART) and the PE platform were developed for military personnel early in their careers, this population represents the target training population. Participants were informed of the VR training and research study that took part as part of their regular training schedule 1 to 2 weeks prior by their commanding officers, who also provided them with Participant Information Statements. Participants were invited to attend multiple PE training sessions over a week in May 2020, June 2021, November 2021, and March 2022. On-base training staff members were also invited to attend open exploration sessions to use PE. A total of 189 participants were involved in the study across all locations (156 ADF trainees and 33 training staff members). Participant numbers for each module differed, as not all trainees were available across all delivery days at each site, and not all modules were tested at all locations (eg, trial week 2 at trial site 2 was shortened due to the COVID-19 lockdown). Furthermore, 12 trainees attended 2 separate trial weeks, and their data were only included in the analysis for their first attendance.

### Ethical Considerations

Research activities were reviewed and approved by the Australian Defense Science and Technology Low Risk Ethics Panel (Protocol Land Division 17-19) and coregistered with The University of Newcastle Human Research Ethics Committee (H-2020-0020). All participants provided written informed consent to participate in this study. Participants were not reimbursed and did not receive compensation for participating in the study. The study was conducted as a scheduled activity within the IET program; however, there was clear communication that participation in the research study was voluntary and outside of their training requirements. An alternative work- or training-related activity, as specified by their commanding officer, was provided to trainees who chose not to participate. A participant ID number was used to match responses where necessary, and no identifiable information (name, rank, or ID number) was collected. Any identifiable data or information provided by a participant within the survey responses were redacted to ensure the anonymity of the participants.

### Study Design

**Figure 2:**
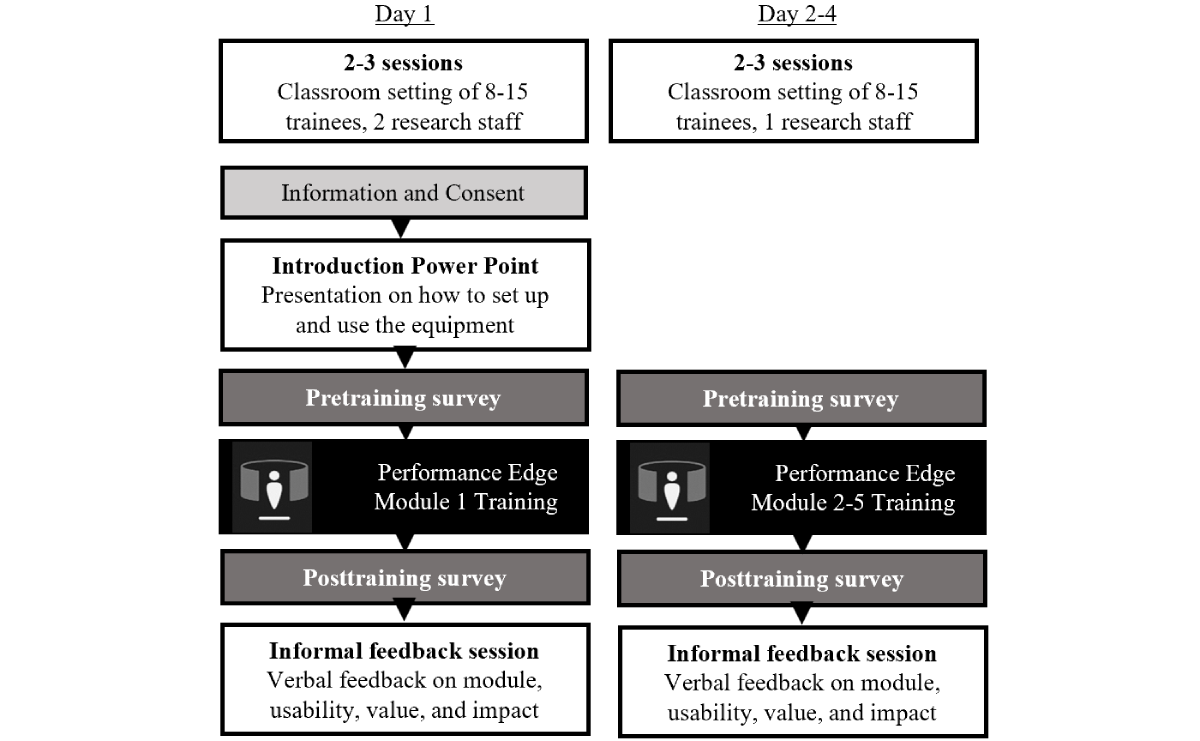
Study design: an overview of approach used for training delivery and surrounding mixed methods data collection.

A mixed methods approach was applied to evaluate the delivery of PE as a multiday classroom-based training program across the 3 training sites. The trainees completed one to five PE modules on consecutive days within a training week. Training and subsequent assessment of each module occurred within a single 1-hour session in a group setting with 8 to 15 participants simultaneously (Figures 1E and 1F and Figure 2).

The trainees completed pre- and posttraining surveys specific to each training module. Each training session was concluded with a 10-minute group discussion focused on providing an opportunity for group feedback.

The on-base training staff attended a single unstructured exploration session, in which any module or component could be explored, and completed the posttraining trainer survey only.

### Measures and Data Collection

This trial employed observational and objective biometric data, as well as self-report and focus group data related to multiple aspects of PE training delivery.

### Self-Report Instruments

#### Training Gains Measures

Each pre- and posttraining survey contained validated scales and general questions on relevant constructs and domains, informed by the technology acceptance model. This included questions to assess technology acceptance (perceived usability, perceived usefulness, and implementation feedback), relevance of content and framework, previous work experience, virtual reality, stress management skills, and perceived training impact. A full list of questions is provided in Multimedia Appendix 1, Table S1.

The study-specific questions were adapted from those previously developed and administered in studies assessing VR training and the first PE pilot study [39]. Questions were constructed using a 3-step process (defining construct, content domains, item generation, and determining the format) from VR experts, teaching, training experts, and ADF members. Questions were drafted by investigator MK and finalized using an iterative approach, with feedback from all study investigators using multiple choice, 5-point Likert scale, or an open-ended format. A list of surveys, validated scales, validity, and relevant references are provided in Tables 2 and 3. All surveys were distributed using QR codes and Office Forms (Microsoft Office).

**Table 2:** Outcome measures—validated scales.

Administered	Name	Internal consistency	Reference
Posttraining for all modules (M1-5)	Modified User Engagement Scale- short form (UES-SF): 12-item tool with 4 subscales designed to measure user engagement with digital domains. Modified Presence Questionnaire (PQ): 29-item scale measuring the subjective experience of being in a virtual environment.	All subscales ω>0.81 (as assumption for α not met) α=.81	O’Brien et al [40] Witmer and Singer [41]
Pre- and posttraining for M3 and M4 only	State Mindfulness Scale: a 21-item self-report measure designed to assess state mindfulness—a person’s present moment attention to their experience during a specified time and context.	α=.95	Tanay and Bernstein [42]
Pre- and posttraining M2 and M3 only	Relaxation Inventory–Physical Assessment: The PAS scale consists of 20 items and quantifies a person’s overall physical state with respect to how relaxed they feel. Relaxation Inventory—Cognitive Tension: 15 items designed to quantify the cognitive tension someone is feeling with questions relating to mental states of worry and anxiety.	α=.95 α=.81	Crist et al [43]

**Table 3:** Outcome measures-developed self-report items.

Administered	Core aspects that were aimed to be addressed	Example question
Pretrial survey (day 1 only)	Preexisting experience with VR^a^ technology and general attitude toward stress management training	Do you have previous experience with VR technology or headset-based simulation technology, either through personal or professional experience?
Pretraining survey specific for each module	Specific preexisting subject matter knowledge and experience	How often do you engage in controlled breathing to manage your stress response? (M2)
Posttraining survey specific for each module	Personal attitudes and feedback on individual components, perceived relevance, skill acquisition, areas for improvement and perceived benefits	Controlled breathing is a useful and effective way of managing stress (5-point Likert scale, disagree to agree; M2)
Posttrial survey (day 4)	Personal attitudes and feedback on the overall Performance Edge training platform, including acceptance of VR technology and general value to ADF^b^ training	How easy was the use of and navigation within the VR tool?

^a^VR: virtual reality.

^b^ADF: Australian Defense Force.

#### Trainer Surveys

Training staff received a tailored trainer survey after exploring PE content. Questions were adapted from trainee surveys and included additional questions on the suitability of the training framework and technology delivery within the broader organization.

#### Observational Data

The research team documented quantitative data, including costs, time frames, hardware charging, and set-up requirements.

#### Objective Biometric Data

Respiratory signals were sampled every 0.05 seconds (20 Hz) and translated into respiratory rate (breaths per minute), calculated after every inhalation. Rates are calculated via a peak detection algorithm using the derivate of data points after smoothing low-pass filtering and are updated after each peak inhalation detection. The average respiratory rate for each 2-minute exercise period was collected and saved, time-stamped, and sent using Wi-Fi to an external cloud-based server with data linked to the serial number of the corresponding headset. Respiratory data integration and collection occurred in modules 2 and 3, respectively. All the modules collected and recorded the selection choices, interactions, and time spent in each exercise.

### Data Analysis

Data from all locations were pooled for analysis unless specifically mentioned in the text, whereas data from trainees and training staff were analyzed and reported separately. Self-reported and objective data were analyzed using Prism (version 8; GraphPad) and JASP (version 0.16.3).

Self-reported data were summarized and presented as mean (SD) or absolute number of responses, and all responses were included. Validated scales were calculated per protocol and analyzed using a 1-tailed parametric test (paired *t* test) or nonparametric equivalent (Wilcoxon signed-rank test), where assumptions were violated.

Respiratory rate data were analyzed using one-way repeated measures ANOVA adjusted for multiple comparisons and a paired-sample *t* test to compare average respiratory rates during training in M2 and average respiratory rates before and after training in M3.

Both *P* values and Bayes factor (BF) have been reported. Values of *P*<.05 are reported as indicators of significance and a BF_10_>3 is considered decisive evidence in favor of a difference or effect.

## Results

### Training Population and Existing Skill Level

Overall, 40% (50/126) reported previous experience with VR technology, largely in the recreational gaming context with 50% (25/50) reporting less than1 hour, 28% (14/50) less than 10 hours, and 22% (11/50) reporting over 10 hours of total exposure. Overall confidence in the set-up and use of VR technology was moderate (mean 3.4, SD 1.2; 1=not at all confident, 5=extremely confident). The remaining 60% (114/126) of trainees had not previously used VR.

Trainees indicated general awareness and theoretical knowledge of stress management, but the perceived utility of specific skills at the outset of training was modest. When questioned about day-to-day attention to their thoughts, emotions, and initial behaviors (“inner world”), trainees reported being very aware (33/64, 52%) or somewhat aware (31/64, 48%) of the degree to which their thoughts and emotions influenced each other and their behaviors. Nearly all participants (62/63, 98%) indicated that they were either very (33/63, 52%) or somewhat (29/63, 46%) aware of the impact that stress can have on their inner world. Of the specific skills taught in PE, controlled breathing was the most highly reported stress management skill used in the cohort pretraining, with 80% of trainees (64/80, 80%) previously engaging in controlled breathing. Although awareness of controlled breathing as an effective stress management skill was high (80/80, 100%; very or somewhat aware), it was primarily applied in a physical and exercise context (eg, target shooting and combat training) rather than for stress management per se. Few other skills and stress management concepts were understood to be effective strategies to reduce stress, and their use was limited. Only 15% (8/54), 23% (23/98), and 36% (15/42) of trainees reported having used progressive muscle relaxation, grounding, or an acceptance strategy to manage challenging emotions, respectively.

### Engagement and Sense of Presence With PE Training

**Figure 3:**
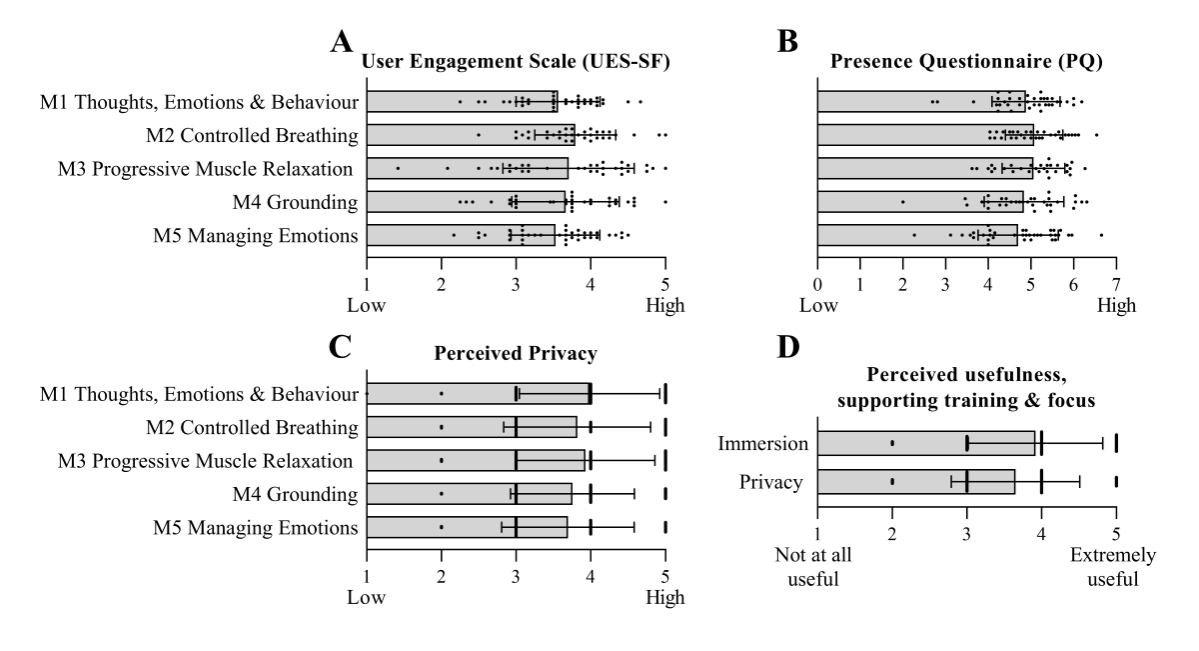
Trainees indicate a high level of user engagement, immersion, and privacy across all 5 Performance Edge modules (M1-5). (A) User Engagement Scale; (B) Presence Questionnaire; (C) Perceived Privacy ratings responses for each of M1-5 administered after each training module (n=29-36); (D) responses to query “How useful was immersion or privacy in supporting the training activities and focus?” (n=61). Responses were on a 5- or 7-point Likert scale for A, C, D, and B, respectively. Data presented as mean (SD).

Engagement with PE training was assessed for each module using the validated User-Engagement Scale–Short Form (UES-SF; [40]) for digital domains, which includes the subdimensions of esthetic appeal, focused attention, perceived usability, and reward. All 5 modules were positively rated by the trainees (Figure 3A), with the highest level of user engagement reported for M2—Controlled Breathing (3.9, SD 0.54) and lowest for M5—Managing Emotions (3.5, SD 0.60; 5-point Likert scale, >3=positive), with no modules scoring below 3.0 (which would indicate a negative assessment) in any UES-SF subdimensions. Across all modules, perceived usability was consistently the highest scoring subdimension.

To assess presence within the experience, the Presence Questionnaire was administered after each training module (Figure 3B). All the modules were perceived as having an overall feeling of presence. The sense of presence ranged from 4.7 (SD 0.94) in M5—Managing Emotions, to 5.1 in M2—Controlled Breathing, and M3—Progressive Muscle Relaxation (PMR) (SD_M2_ 0.67; SD_M3_ 0.73; 7-point scale). The single item assessing perceived privacy was rated above 3.6 for each module (5-point scale, Figure 3C).

By design, all training modules and most exercises within PE contain interactive components, including actions and response options for trainees. Trainees responded to interactions within 6 to 31 s, with response times varying by the number of response options, complexity, and familiarity with the exercise. Although answer options were never considered “correct” or “incorrect,” certain exercises and scenarios aimed to invoke a particular emotion or thought response. For example, 87% (32/38) of trainees selected either relaxed or happy as the main emotion felt during an intentionally relaxing 360° video beach environment in M5—Exercise 1.

In an open-ended response section, immersion and privacy of VR technology were named the most beneficial components. This observation was supported by additional question responses, in which both immersion and privacy were rated to be useful or extremely useful in supporting training and attention focus (immersion=3.9, SD 0.90 and privacy=3.7, SD 0.86, respectively; 1=not at all useful and 5=extremely useful; Figure 3D). Biofeedback functionality, interactive elements, and concept visualization were also named as particularly positive features of PE training across all modules in open-ended questions. Survey responses consistently provided positive feedback on individual modules while endorsing the overall length of individual exercises.

When asked which elements of the training could be improved, open-ended responses primarily included the expansion of existing elements, escalation of provocative content and scenarios, and inclusion of ADF-specific content.

### Module-Specific PE Training Outcomes

**Figure 4:**
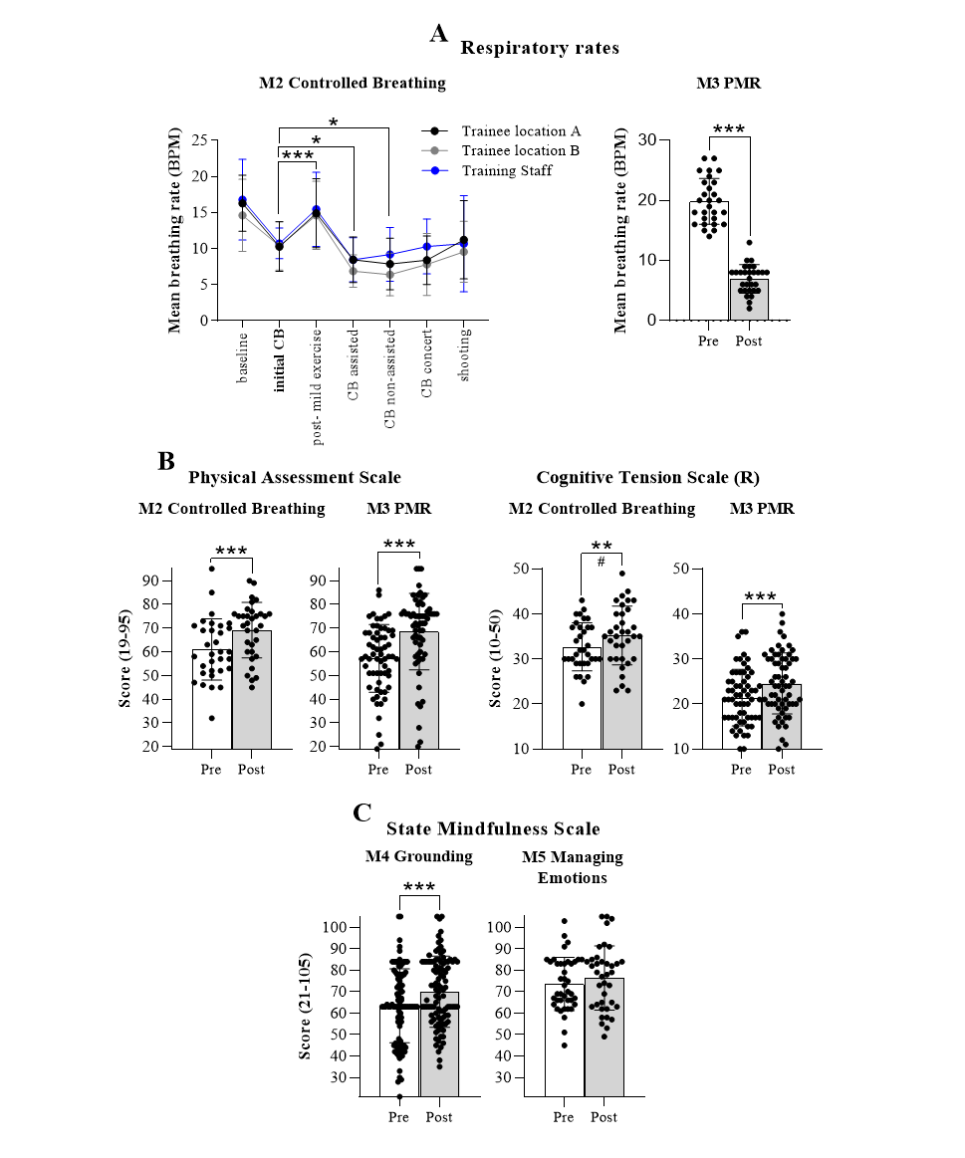
Objective measures of training impact for modules 1-5 (M1-5). (A) Mean breaths per min (BPM) and standard deviation collected during Controlled Breathing (CB) training (M2) and Progressive Muscle Relaxation (PMR) training (M3) for trainees and training staff across all training exercises of M2 as well as pre and post the M3 guided exercises. Repeated measures, one-way ANOVA followed by Dunnett multiple comparison test to initial controlled breathing. PMR trainees n=27, 1-tailed paired *t* test. (B) The relaxation inventory reported as separate subscales, physical assessment scale (PAS) and cognitive tension scale (CTS). Scales were collected pre- and post-M2 and M3 training. M2 trainees n=33; M3 trainees n=31; 1-tailed paired *t* test for M2 PAS scores. One-tailed Wilcoxon signed-rank test for M3 PAS, and M2/3 CTS scores. R=denotes reverse scoring. (C) State Mindfulness Scale collected pre- and post-M4 (Grounding) and M5 (Managing Emotions) training. One-tailed Wilcoxon signed rank tests. M4 trainees n=98, M5 trainees n=35. All data points presented (including outliers), graphs presented as mean (SD), **P<.01; #BF10>22; ***P<.001, BF10>100.

Module-specific training perceptions were assessed using the objective and qualitative outcomes relevant to the specific training objectives of each module. The central training objective within M2—Controlled Breathing, was to gain awareness and control over breath cadence, specifically to maintain a slow and steady breathing rate across escalating training exercises (Figure 4A). Consequently, changes in breathing rates were used to gain insights into training outcomes with the hypothesis that exercises within PE would result in a reduction in breaths per minute (BPM) compared with the initial, noninstructed breathing rates recorded at the start of the module. Initial baseline respiratory rates, without prompts, were 16.3 (SD 3.9) breaths BPM for trainees and 16.8 (SD 5.6) BPM for training staff. When prompted to actively reduce their breathing rate, initial mean breathing rates reduced to 10.3 (SD 3.4) BPM (trainees) and 10.7 (SD 2.1) BPM (staff). Live biofeedback with a respiratory trace (termed “controlled breathing assisted”) supported further reductions to 8.5 (SD 3.2) BPM (trainees) and 8.5 (SD 3.1) BPM (staff). Participants subsequently sustained reduced controlled breathing rates without live biofeedback (“controlled breathing non-assisted”), and in subsequent exercises incorporating a distracting environment (“controlled breathing concert”) and shooting tasks. Training effects for M3—Progressive Muscle Relaxation were assessed using changes in breathing rates and state relaxation before and after the 15-minute guided relaxation exercise. Mean respiratory rates were significantly reduced compared with baseline at the start of the exercise, from 19 (SD 3.8) to 6.9 (SD 2.3) BPM (mean difference −12.74, SD 3.4; *t*_26_=19.198, *P*<.001; BF_10_=1.413e+14).

The Physical Assessment Scale (PAS) and Cognitive Tension Scale (CTS) subscales of the Relaxation Inventory were administered before and after both M2 and M3 to quantify relaxation states (Figure 4B). Owing to the common use of controlled breathing and PMR for relaxation, we hypothesized that training would result in an increased self-report rating on the PAS and reduced ratings on the CTS. As hypothesized, physical relaxation (PAS) significantly increased after both M2—Controlled Breathing (mean increase 7.78, SD 9.9; *t*_30_=−4.354, *P*<.001; BF_10_=374.435) and M3—PMR training (mean increase 11.27, SD 14.49; w=224, *Z*=−5.087; *P*<.001, BF_10_=891.313). Similarly, cognitive tension scores (reverse scored to indicate relaxation) increased after both modules (M2 mean increase 2.53, SD 6.04; w=87, *Z*=−2.451*,* *P*=.007; BF_10_=22.523; M3 mean increase 3.4, SD 5.10; w=193, *Z*=−4.406, *P*<.001; BF_10_=3543.625).

Given the training objectives for M4 and M5, we explored the assumption that training may increase mindfulness, as measured using the State Mindfulness Scale (Figure 4C). M4—Grounding increased mindfulness scores from 63.42 (SD 17.26) to 69.98 (SD 16.57) , a significant change (mean increase 6.14, SD 15.22; w=906.5, *Z*=−4.036, *P*<.001; BF_10_=5067.078). A trend toward increased State Mindfulness Scale scores following M5—Managing Emotions training was not statistically meaningful (mean increase 2.97, SD 12.45; w=155, *Z*=−1.351, *P*=.09, BF_10_=0.743). This was true for both the Body (mean increase 0.50, SD 5.195; w=120.000, *Z*=−1.410, *P*=.08; BF_10_=0.830), and Mind (mean increase 2.47, SD 8.97; w=186.5, *Z*=−1.449, *P*=.08; BF_10_=1.452) subscales. However, the Bayes factor for the Mind subscale (BF_10_=1.452) provided anecdotal evidence of an increase in mental-state mindfulness.

**Figure 5:**
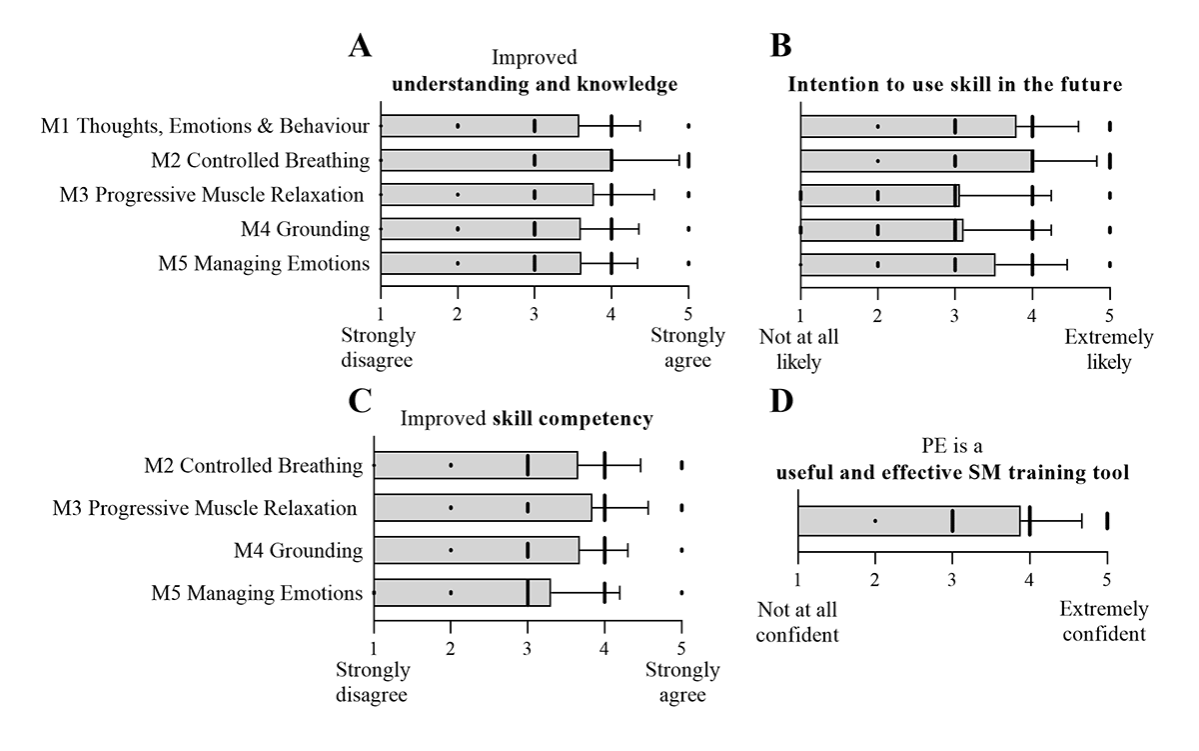
Self-reported training impacts on knowledge, skill competence, intended future use and overall training effects. Responses on 5-point Likert scales were collected after each module (M1-M5) presented as mean (SD) (1=strongly disagree or not at all likely; 5=strongly agree or extremely likely). (A) Improvement of understanding and knowledge; (B) future use of the skills; (C) improved skill competency. Response numbers: M1=69, M2=76, M3=56, M4=104, M8=36. Responses collected at the last day of a training using a 5-point Likert scale; n=113 (1=not at all confident; 5=extremely confident) for (D) Usefulness and effectiveness of the platform as a stress management training tool. PE: Performance Edge.

In addition to objective outcomes, self-reported data on training impact, efficacy, and perceived value were collected after each module and final training session. Following training, trainees reported a deeper understanding of the theoretical concepts taught within PE, specifically for underlying practical skills (M1: 3.9, SD 0.7; M2: 3.8, SD 0.7; M3: 3.8, SD 0.71; M4: 3.8, SD 0.7; M5: 3.8, SD 0.7; 1=strongly disagree; 5=strongly agree; Figure 5A). Perceived skill competency improved after training for M2—Controlled Breathing, M3—PMR, M4—Grounding and M5—Managing Emotions (M2: 3.7, SD 0.8; M3: 3.8, SD 0.7; M4: 3.7, SD 0.6; M5: 3.3, SD 0.9; 1=strongly disagree; 5=strongly agree; Figure 5C). Statistical analysis was conducted on self-report items asking about the likelihood of engaging in these skills. We hypothesized that PE training would increase the likelihood of engaging in the respective skill in a stressful context. Trainees indicated they were more likely to actively consider their thoughts and emotions before reacting to a stressful event (mean 3.8, SD 0.8 on a 5-point Likert scale; mean difference [post-pre]: 0.46, SD 0.7; Wilcoxon signed-rank test *Z*=−2.548, *P*=.002; BF_10_=27.94). Similarly, trainees were more likely to use grounding skills after PE training compared with pretraining (mean increase 0.55, SD 0.9; Wilcoxon signed-rank test *Z*=−2.353, *P*=.008; BF_10_=9.118). Although the likelihood of using other skills did not change pre-post training, trainees indicated they were likely to engage in controlled breathing (4.0, SD 0.8) and use an acceptance strategy to manage their emotions (3.5, SD 0.9) the next time they encountered a stressful or challenging event (1=not at all likely, 5=extremely likely; Figure 5B), indicating that pretraining levels were already favorable toward these skills. The overall intention to use PMR and grounding skills after completing M3 and M4 training was only modest (mean 3.1, SD 1.2 and 3.1, SD 1.1, respectively). Overall, 66% (75/113) of participants were confident or extremely confident that PE represented a useful and effective platform to train and practice stress management skills (3.9 SD, 0.8, 1=not at all confident; 5=extremely confident, Figure 5D), whereas only 2 of 113 trainees stated they were not confident that the platform was a useful stress management training tool.

### Trainer Feedback

**Figure 6:**
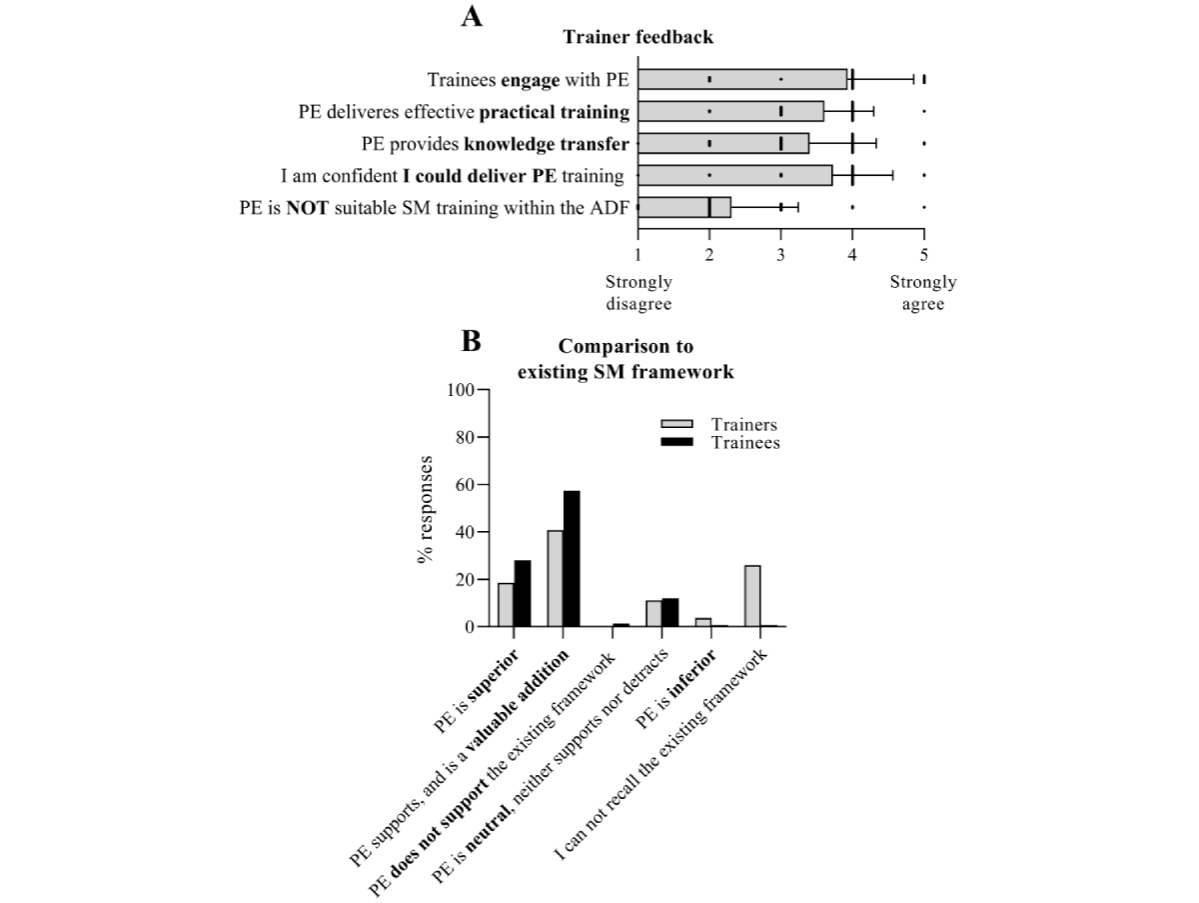
Trainer feedback on Performance Edge. (A) Training staff perceptions of PE. Answers given on 5-point Likert scale (mean, SD; n=28). (B) Training staff and trainee responses on alignment of PE with existing SM training within the ADF. Single response, multiple-choice question (Trainees n=150, Trainers n=27). ADF: Australian Defense Force; PE: Performance Edge; SM: stress management.

Most trainers agreed or strongly agreed that trainees engaged with the training content (24/29, 83%) and that the platform delivered effective practical training on stress management skills (18/28, 64%; only 2 trainers disagreed). Furthermore, 50% (15/30) of the training staff agreed that the platform provided valuable knowledge transfer, whereas 5 disagreed with that statement (Figure 6A). Importantly, 79% (23/29) of the trainers stated that they were confident or extremely confident in their ability to deliver VR training in the classroom. Verbal feedback and responses to an open-ended question indicated that staff members were positively surprised by the platform and how the technology supported fundamental skill development. With expected stress inoculation training, many saw great benefits in approaching cognitive and emotional skills training within the immersive and private environment of the VR headset. Engaging and interactive components were named particularly beneficial and contrasted with the traditional delivery approach using PowerPoint-based materials.

Trainer quotes from survey:

[Performance Edge modules are] really good at helping people identify their responses to situations.

It is very beneficial to include practical training related to a soldier mindset.

Although trainers praised the use of nonmilitary design, language, and introduction segments, both trainees and trainers suggested that the final exercises in each module would benefit from being placed within a relevant military context.

#### Alignment of PE With the Existing ADF Training Framework

The PE platform was developed as an extension of the ADF stress management framework. Compared with the existing approach, most staff and trainees indicated training added value or was superior (Figure 6B). Specifically, for staff, 41% (11/27) believed PE to be a valuable addition, which supports existing stress management training, and 19% indicated that PE was superior (a combined total of 60%). Of the remaining staff, 26% indicated that they were unable to recall the existing stress management training and, therefore, were unable to make a comparison, 11% felt it neither supported nor detracted from existing training, and only 1 trainer (out of 27) indicated that PE was inferior. For trainees, 57% (86/150) believed PE to be a valuable addition, supporting existing stress management training, and 28% indicated that PE was superior (combined total of 85%). Moreover, 12% felt that it neither supported nor detracted, and only 1 trainee (of 150) indicated that it was inferior to the existing approach.

#### Delivery of PE Training

Data on trial logistics and requirements were also collected to inform the implementation strategies and determine their feasibility. A total of 372 PE training sessions were delivered over 9 days across 4 training weeks. Average “in headset” training time (in minutes) for each module was M1=22.9 (SD 4.2), M2=17.4 (SD 2.8), M3=18.9 (SD 1.8), M4=33.9 (SD 1.3), and M5=32.6 (SD 6.2).

Content was loaded on headsets off-site before each trial week (duration approximately 20 minutes) and the initial on-site hardware set-up of 20 headsets required approximately 1 hour by a study team member. A full headset charge from 0% to 100% required approximately 3 hours. After three consecutive training sessions (approximately 1.5-hour active runtime plus standby time of approximately 5 h), the average headset battery charge was 40%. The hardware was charged overnight between training days. A silicone cover was used over the headset face foam. The silicone cover, outside the headset, controllers, and respiratory belt were wiped down before and after each session using a skin-friendly VR head mounted display cleaning wipe provided to the trainees (a typical classroom set-up is shown in Figure 1E).

Trial weeks in 2021 occurred under COVID-19 social distancing requirements (1.5 m distancing between individuals; Figure 1F). Although the first session each week required specific instructions and clarifications on how to set-up, use, and navigate the hardware, trainees required limited input in subsequent sessions. Additional instructions were provided for the biofeedback-enabled modules to ensure that the respiratory belt was plugged into the headset. Trainees required repeated and specific instructions on how to re-enter the VR field of view. Attendance by two instructional staff members to support the delivery was useful, but only required during the first session each week to support information and consent processes for the research component of the trial. For standard operational training delivery outside the research context, one team member would be sufficient.

In addition, 98% (363/372) of the training sessions were delivered without any issues or difficulties identified. During the initial March 2020 trial (n=42 total participants), a total of 8 technical issues were reported. All but two technical issues were resolved with assistance during the session. The remaining two issues related to automated data capture were not apparent until the completion of the training. Five technical issues related to the connection between the respiratory belt and the VR headset (specifically, the USB-C wired connection). The USB-C connection issue was resolved for subsequent trial dates by replacing the USB-C adapter with a robust model. The remaining issues included freezing of the VR headset screen, operator difficulties, and set-up of the VR “guardian area.”

During the November 2021 trial (n=37 participants), 7 trainees were unable to complete the final exercise of M1 owing to a loading issue in the software, which was subsequently addressed and resolved for the March 2022 trial. Two additional trainees accidentally exited the training module. No VR-induced motion sickness was reported directly or in the survey responses during or after training. The research team was informed of a single unanticipated response to one exercise in the M4—Grounding module, in which a trainee reported to the ADF chaplain that the memory recall component of the training triggered emotional distress. As a result, a warning was included in subsequent versions, noting the potential for an emotional response.

## Discussion

### Principal Findings

This trial describes the delivery of the PE prototype as a VR-based practical training platform for fundamental stress management skills within a workplace setting. The outcomes demonstrated the perceived usefulness, feasibility, usability, and positive training outcomes of the technology platform, training concept, and specific training modules within the intended real-world context and training population.

PE was delivered to 189 military trainees during consecutive 1 hour in-classroom training sessions of up to 20 trainees at a time and 5 modules in total. The distribution and utility of the biofeedback-integrated VR system were portable, easy to set-up, and suitable for the needs and requirements of the training organization and the target training population. Both the intended training population and their training staff perceived the platform to be useful, easy to use, engaging, immersive, and aligned with the existing stress management training framework (Figures 4 and 6). Based on the technology acceptance model, our results for perceived usefulness, immersion, and engagement suggest that future adoption of the platform is highly feasible [44,45]. The ability to practice cognitive strategies in a diverse, private, and immersive training environment, while in a group setting, was highlighted as particularly valuable and supported the training objectives. Training benefits were observed in both physiological and mindfulness outcomes for specific training modules (Figure 4). Consistent positive feedback and self-report responses from both the training staff and trainees indicated increased knowledge, skill competency, and intention to use certain skills in the future (controlled breathing, awareness, and emotional acceptance; Figure 5).

### PE Is a Feasible VR Training Solution for Group-Based Training

VR technology is an emerging field of research in military contexts and is predicted to improve training effectiveness in multiple domains, including combat command and decision making [35,46,47]. The use of VR as a training modality is relatively new, and very few training organizations have sustainably adopted, scaled, or integrated technology within their training continuum. This is particularly true for VR-based cognitive and psychological stress management interventions as their integration into the workforce has been challenging [14,48,49]. The relative novelty of VR-based stress management training has resulted in research focusing largely on the efficacy of applications under controlled experimental conditions [50]. However, these types of controlled studies cannot address questions about feasibility and implementation in real-world contexts, and there are limited reports on implementation challenges in the literature [51]. Factors that may impair the uptake of an otherwise effective digital tool include technology acceptance, practical usability, proficiency in use, and the existing structures required to support the technology [52]. This was due to the paucity of information on implementation issues, which we placed a particular emphasis on in this study.

After our original study evaluating PE in a controlled research trial, we found that the initial set-up and hardware was impractical, complex, and unstable outside of a research setting, particularly the research grade biometric data collection system. The platform underwent significant redesign to address challenges related to practical implementation within a group-based classroom setting [39]. We demonstrated the effective delivery of training to groups within their existing unit size, using a trainer to trainee ratio of 1:20. Seamless delivery was made possible using a freestanding headset solution and an enterprise hardware version, allowing remote fleet management, software upload, and updates. Familiarization with the technology occurred quickly despite limited previous trainee experience with VR technology. Notably, no occurrence of motion sickness, cybersickness or dizziness, a common concern among first-time VR users, has been reported [53,54]. Taken together, the results from this trial suggest that PE represents a VR training solution that is suitable for group-delivered training in the workplace context.

### Engagement and User Acceptance of a Novel Training Solution

PE represents a novel training approach, not because of the intrinsic strategies included within the platform but rather by delivering practical training in a diverse, engaging, and immersive training environment that is private despite delivery in a group setting.

An important feature of PE is the emphasis placed on the practical development of stress management skills [55]. Consistent with BattleSMART, PE adopts the perspective that stress-management skills are central to optimal human performance. Throughout the modules, language with an overt mental health tone has been avoided. Instead, the perspective is taken that stress management skills should be considered like any other skill that contributes to healthy rounded performance in the workplace. Throughout the modules, the trainee is encouraged to consider stress management skills as general life skills that can be usefully applied not just to major stressors but also in response to small or mundane day-to-day challenges. The effort to recast validated skills within the platform was broadly received by the training audience.

In addition to the practical challenges of implementation, critical predictors for the future use and acceptance of new technologies in education are perceived usability and usefulness [44]. User engagement is particularly relevant for the transition of digital mental health interventions into real-world practice is user engagement [56,57]. However, transitioning from an expert-led to a digital-training format is often associated with low engagement, shallow learning, and potential frustration [51,58]. Given the technological and framework novelty of PE as a training solution for stress management soft skills, critical elements related to technology acceptance were investigated in this case study.

User engagement was validated for the target training population, specifically for military staff undergoing initial employment training. Feedback from both trainees and trainers suggests a general level of enjoyment and high levels of engagement for all modules (Figures 3A and 6A). Engagement is further supported by self-report ratings on the UES-SF and its subscales (engagement, esthetic appeal, focused attention, and perceived usability) as well as data collection and response times within the headset, which indicate that users participated in the activities, including personal reflections, as intended. Although the UES-SF is not intended to compare scores across applications or empirically classify high and low ratings, positive ratings and general training participation provide evidence of end-user acceptance of the training platform [40].

Immersion and sense of presence within PE are important to validate, as 360° video and computer-generated interface activities were intentionally designed to generate relaxing (beach and forest scenes), interesting (at an aquarium, in a sports gym), distracting (at a rock concert), or confrontational (angry men) training environments. The terms “immersion” and “presence” are often used interchangeably. However, in the scientific literature, presence refers specifically to the subjective psychological response of being within the environment, whereas immersion describes objective inputs within the digital environment (eg, interactions with the surroundings and selection items) [59,60]. The cognitive skills and reflections practiced within PE benefit from a sense of presence, as research suggests that presence can prompt emotional responses and interactions with digital avatars and environments despite users being fully aware of the fictitious nature of the setting [61-63]. A sense of presence has also been linked to improved training efficacy in digital-training applications [64,65]. All modules of PE generated a sense of presence (average rating of 5 on a 7-point scale; 7=high, 0=low), and the training environments triggered emotional states as intended (Figure 3). In support of VR technology, immersion and privacy were rated as valuable and specifically mentioned as useful elements of the platform (Figure 3). An important element connected to trainee engagement with the subject matter (specifically, emotional, thought awareness, and memory recall activities) was the affordance of privacy in a group setting provided by the VR headset. Existing research into the use of VR in education and training has shown the benefits of immersive VR over 2D screen delivery in areas of increased relaxation and arousal, motivation, engagement, and interest [34,66-68]. Although future research is required to validate the outcomes for PE platform in direct comparison to a screen-delivered training tool, it seems unlikely that trainees would feel an equivalent sense of presence, privacy, and engagement in a room and training setting of 20 trainees.

### Training Outcomes

This study demonstrates the perceived usefulness of PE training, directly and indirectly, through self-report of improved skill competency and enhanced knowledge for each module (Figure 5). Both training staff and trainees reported that PE provided useful and effective practical training and represented a valuable addition to the existing program, particularly via the provision of biofeedback, as well as the use of immersive and interactive training components. In pretraining surveys, ADF members indicated a relatively high level of understanding of the benefits and familiarity with concepts related to stress management skills. These findings are consistent with reports of military personnel who respond positively to the use of stress management techniques [69]. This background knowledge is likely to be, at least in part, due to previous exposure to the ADF BattleSMART program, which provides comprehensive education on optimal emotional and behavioral outcomes, resiliency, and arousal reduction skills [31]. However, despite this existing awareness, ADF staff reported minimal application of stress management skills outside of the training. Trainees had heard of grounding and progressive muscle relaxation but had very little practical experience in using them.

Modules 2 and 3 (controlled breathing and progressive muscle relaxation) aimed to develop skills that reduce the physiological effects of stress. The training outcomes included improved relaxation states and reduced respiratory rates (Figures 4A and 4B). Trainees were able to effectively reduce their respiration during the controlled breathing module, which is comparable with the results of our previous pilot trial [39]. Gaining conscious control, reducing breathing rates, and increasing relaxation are elements associated with effective stress management and reduced stress [70,71].

Modules 4 and 5 (grounded and emotional acceptance) provide training on cognitive skills; thus, skill competency and training outcomes for these modules were difficult to objectively quantify and compare with existing training approaches. There is growing literature on the relationship between mindfulness, psychological health, and stress reduction [72]. As elements of grounding overlap with mindfulness, the State Mindfulness Scale was administered, and an increase was observed following module 4 training. Although no differences between pre- and posttraining mindfulness states were observed for module 5, this may be due to the specific items used within the State Mindfulness Scale. Items within the body subdomain may not be relevant to training in emotion identification and acceptance. Although increased state mindfulness suggests a beneficial impact on stress, further research would be useful in assessing the efficacy of these training modules (M4 and M5).

Overall, the data suggest a positive immediate impact of PE training across the four stress management skill areas. Further studies will be useful to assess whether these short-term effects translate effectively to skills consolidation, the application of skills to real-world contexts, and the long-term effects of behavior changes and stress outcomes.

### Study Limitations

The current trial was intended to assess the usability, feasibility, and suitability of PE training within its target training population in a real-world context. It should be noted that this is a case study within the ADF, and thus the findings may not be generalizable to other training settings, user populations, or training tools. Given the limited amount of investigation into the effective implementation of VR technology in the workplace, the results of this study provide valuable information on how to effectively integrate VR training into the workplace. Although specific to the ADF, we would view this as a useful starting point for any large organization interested in using VR within their training continuum. This work is intended to be the first step, with future studies required to document training efficacy. In particular, future research should investigate the number of iterations of VR exposure to develop skill mastery, the rate of skill degradation, how effectively these skills can be incorporated into real-world performance, and any effects on long-term mental health outcomes and workplace performance. Software and hardware were updated iteratively in response to the identified issues across the project and differed slightly between trial locations. To mitigate self-report bias, a conscious effort was made to brief participants that their input and responses were completely anonymous and that their input was being sought to improve the development of the application. Unfortunately, not all of the modules could be tested at all trial locations owing to staff availability and last-minute changes resulting from COVID-19 restrictions. As a result, trainee response numbers varied across modules (all numbers are reported). Despite this limitation, the multilocation trial approach resulted in participant numbers that far exceeded numbers generally seen within the VR literature, provided consistent findings for ADF service members across varying branches and locations, and provided balanced feedback for this level of evaluation.

### Conclusions

This study found that the PE platform was feasible, implementable, and acceptable for stress management skills training within the ADF. Although many other studies have only assessed training solutions in controlled study environments, our work shows that virtual-reality and biofeedback technology can support training in real-world workplace settings. The ability of the PE platform to generate a private and immersive environment within a group setting provides a valuable proposition for the use of VR for this type of cognitive training. Engagement with the training platform is likely connected to the use of a targeted training framework as well as an approach and philosophy that is aligned with the overarching organizational values and practical requirements.

## Appendix

Multimedia Appendix 1 Additional information and self-report items.

Multimedia Appendix 2 Promotional video for the Performance Edge software recorded within the headset (it is advised that the recording may result in motion sickness).

Multimedia Appendix 3 Module 1 walk-through start. This video was recorded as a screen capture within the virtual reality headset and shows the beginning of module 1 (thoughts, emotions, and behaviors). It is advised that the recording may result in motion sickness.

## Abbreviations

ADF: Australian Defense Force
BF: Bayes factor
CTS: Cognitive Tension Scale
IET: initial employment training
PAS: Physical Assessment Scale
PE: Performance Edge
PMR: Progressive Muscle Relaxation
SM: Stress Management
UES-SF: User-Engagement Scale–Short Form
VR: virtual reality

## Data Availability

The datasets generated and analyzed in this study are available from the corresponding author upon reasonable request.
