# Correction: Evaluation of a Virtual Reality Platform to Train Stress Management Skills for a Defense Workforce: Multisite, Mixed Methods Feasibility Study

**DOI:** 10.2196/54504

**Published:** 2023-11-20

**Authors:** Murielle G Kluge, Steven Maltby, Caroline Kuhne, Nicole Walker, Neanne Bennett, Eugene Aidman, Eugene Nalivaiko, Frederick Rohan Walker

**Affiliations:** 1 School of Biomedical Sciences & Pharmacy Faculty of Health, Medicine & Wellbeing The University of Newcastle Callaghan Australia; 2 Centre for Advanced Training Systems Faculty of Health, Medicine & Wellbeing The University of Newcastle Callaghan Australia; 3 School of Psychological Sciences College of Engineering, Science and Environment The University of Newcastle Callaghan Australia; 4 Army School of Health Australian Defence Force Canberra Australia; 5 Joint Health Command Department of Defence Canberra Australia; 6 Human and Decision Sciences Division Defence Science & Technology Group Edinburgh Australia

In “Evaluation of a Virtual Reality Platform to Train Stress Management Skills for a Defense Workforce: Multisite, Mixed Methods Feasibility Study” (J Med Internet Res 2023;25:e46368) the authors made an amendment.

The *Acknowledgments* section:

This study was funded by the Australian Department of Defense through the Defense Innovation Hub open-submission process. We would like to acknowledge all members of the Australian Defense Force who contributed to and participated in the studies, university staff members, and software developers at JumpGate who assisted with study support and implementation.

has been amended as follows:

This study was funded by the Australian Department of Defence through the Defence Innovation Hub open submission process. We would like to acknowledge all members of the Australian Defence Force who contributed and participated in the studies, especially our primary project manager Caroline Ellis. We would like to acknowledge significant contributions made by Dr. Elizabeth Ditton and Brendon Knott, Clinical Psychologists, who contributed to the development of content for the Performance Edge platform. We thank Centre for Advanced Training Systems members, particularly Ann Stevenson and Adam Niesler for constructive feedback and ideas during development and internal testing and trial support. Finally, we thank all companies involved in content creation of Performance Edge including Jumpgate VR, eluminate creative and Glenn Edwards.

The correction will appear in the online version of the paper on the JMIR Publications website on November 21, 2023 together with the publication of this correction notice. Because this was made after submission to PubMed, PubMed Central, and other full-text repositories, the corrected article has also been resubmitted to those repositories.

