# Supplementary material for: Evaluation of a Virtual Reality Platform to Train Stress Management Skills for a Defense Workforce: Multisite, Mixed Methods Feasibility Study
Source: J Med Internet Res. 2023 Nov 6;25:e46368. doi: 10.2196/46368 (PMC10659241; doi:10.2196/46368)
Supplement: Multimedia Appendix 1 [file jmir_v25i1e46368_app1.doc]

Evaluation of a Virtual Reality Platform to Train Stress Management Skills for a Defense Workforce: a Multi-site, Mixed Methods Feasibility Study.

Murielle G. Kluge^1,2^, Steven Maltby^1,2^, Caroline Kuhne^1,3^, Nicole Walker^4^, Neanne Bennett^5^, Eugene Aidman^2,6^, Eugene Nalivaiko^1,2#^ & Frederick Rohan Walker^1,2#*^

^#^ contributed equally as senior authors

**Supplementary Information:**

1. **Performance Edge Training Framework and Concept:**

The training framework and theoretical background for Performance Edge is an extension of and build on the concepts within BattleSMART, the current stress management/resiliency framework used in the Australian Defence Force (ADF). Content is derived from cognitive behavioural therapy principles (CBT) with additional aspects from Acceptance and Commitment Therapy (ACT) and sports psychology.

Concept and content scripting for each module commenced in 2018 in close collaboration with the Office of Strategic and Operational Mental Health, Joint Health Command, ADF and clinical psychologists Brendon Knott and Elizabeth Ditton.

The general training framework and messaging used in Performance Edge is structured as follows:

1. **Stress is a common component of life**. Throughout your professional and private life, you will be exposed to high stress events, including acute and chronic stress events and those which are common and specific to you and your occupation. Like any other skill you learn throughout your basic training, **an optimal response to a stressful event can be trained** and you can practise your reaction to ensure optimal performance and outcomes. Performance Edge is designed to provide targeted training to help you respond to stress effectively.
2. **Stress (chronic and acute) affects us on 4 different levels** (=4 core domains): Initial physiological reactions (of your body), initial thoughts, initial emotions, and initial behaviours. These domains are strongly connected but distinct. Performance Edge training is focused on addressing stress effects within individual domains.
3. The first step towards an optimal stress response is the ability to **identify the effects of stress within yourself** (ideally within all 4 core domains)**.** Performance Edge contains educational components and practical exercises on how to identify and distinguish the effects of stress within the 4 core domains. A distinct emphasis is made on the relationship between thoughts, emotions, and behaviours.
4. Once you are aware of the effects of stress you can utilise strategies to address and manage them. This allows you to:

a) **harness the helpful effects of stress**,

b) **prepare effectively** for a stressor,

c) **make better behavioural choices**,

d) **engage the appropriate stress management strategy** at the appropriate time and

d) **recover quickly** after a stressful event.

Performance Edge emphasises practical training of skills targeting the physiological effects of stress and cognitive strategies associated with managing unhelpful effects of stress on thoughts and emotions.

# Structure: modular training of fundamental skills

This Performance Edge version contains 5 training modules each addressing a distinct training objective and skill. All training modules contain a general introduction and exit video surrounding multiple escalating practical exercises and are approximately **25min in length**. Introduction videos (2min) cover the general concept of the practical skill or cognitive strategy. Exit videos (30sec) summarise the core training messages and outcomes whilst encouraging reflection on areas where the skill may be personally useful to the trainee. To emphasize the use of the skills in any context training occurs largely in an abstract setting and framework. VR training was accompanied with real life facilitated de-briefing session in which trainees are encouraged to discuss contexts where they view a learnt skill would be useful to apply. This means trainees contextualise the skill themselves rather than the training postulating where and when to use it. Examples in which the deployment of the skills is effective are given throughout, are featured in the introduction and final exit section prior to and after the practical skills training in VR.

1. **Design philosophy and elements:**

Performance Edge includes validated skills and techniques, including grounding, PMR, emotional acceptance exercises and controlled breathing, which are commonly used in a mental health context or for secondary prevention of stress. Importantly, Performance Edge reframes these skills and stays away from any mention of a mental health or pathology. Instead, the platform uses a tone, terminology, design, and delivery modality that is aligned with the philosophy that these skills are fundamental life skills and can be useful in any challenging situation. By doing so the program takes on a preventative or prospective approach to stress management.

Performance Edge adopts a futuristic design, which pays homage to space-themed computer games. While the target audience is military, visual design features were created using a neutral pallet without specific reference to a military design, situations, or terminology. This was in part due to the military tri-service specific branding but also allowed the emphasis that the skills can be relevant for any stress-provoking situation, either work-related or in everyday life. Alignment with the ADF values was generated by using a clear and directive tone for instructions, feedback, and explanations with an emphasis on practical elements. All exercises were designed to take advantage of the immersive nature and interactive capability of VR technology to create an engaging learning and training environment. 360-degree scenes are used in the platform to generate a backdrop that is either relaxing (beach and forest scenes), interesting (at an aquarium, in a sports gym) or distracting (at a rock concert). Interpersonal conflict and confrontational scenes utilise 360-degree videography and actors directly addressing the viewer/ trainee to generate a challenging or uncomfortable event for training purposes. For example, Module 4 contains an exercise containing a 360- degree video of a verbal altercation in a bar. After the rather aggressive individual accuses the trainee of being rude and walks away the trainees remain in the bar scene and are instructed to use grounding techniques to calm down. Challenging situations, particularly annoying and frustrating elements are generated by instructing trainees to perform CGI games and activities. For example, a box stacking activity in Module 5 has escalating levels. The first 2 levels are relatively easy, enjoyable, and rewarding with positive feedback and clean UX and graphics. Levels 3-5 dramatically jumps in difficulty whilst also introducing purposeful glitches, rigged outcomes and negative/ harsh feedback. This activity is designed to generate an environment for trainees to notice and name emotions and reflect on the context and underlying values that may have contributed to this emotional response.

Reflections during and after challenging events are supported by the inclusion of guided narration, however the platform encourages and ensures participation by making trainees actively select responses. For example, after a verbal altercation scene in Module 1 trainees chose from a list of options how they would like to respond / what they would like to do next and are then instructed to reflect on the emotions and thoughts that have contributed to this choice, again by selecting the most likely options from a list. The use of interactive elements is particularly relevant for cognitive skills of emotional awareness and grounding trained within the platform. Importantly, the selection of thoughts, emotions or behavioural responses are only used to facilitate in depth reflection and participation and not defined as “correct” or “incorrect” or part of the feedback provided.

**Supplementary video(s):**

***Supplementary video 1: Promo video***

This video contains a short promotional video for Performance Edge recorded within the headset. It is advised that the recording may result in motion sickness.

***Supplementary video 2: Module 1 walkthrough***

This video was recorded as a screen capture within the VR headset and contains the beginning of Module 1 (Thoughts, Emotions and Behaviours). It is advised that the recording may result in motion sickness.

**Supplementary Table S1: Self-report Questions**

| **Pre-Training Questions for Trainees** | | |
| --- | --- | --- |
| **Question** | | **Response option** |
| Q1 | What is your current Rank/Position? | Open-end response |
| Q2 | Have you previously completed Battle SMART training? | Y/N |
| Q3 | How long ago did you complete Battle SMART training? | Open-end response |
| Q4 | How aware are you of different techniques of stress management? | 5-point scale from 1=“not at all aware” to 5=“extremely aware” |
| Q5 | Do you have previous experience with VR technology or headset-based simulation technology, either through personal or professional experience? | Y/N |
| Q6 | How long have you spent directly engaged with VR? | Multiple choice |
| Q7 | Please rate how confident you are with the use of VR in a general context? | 5-point scale from 1=“not at all confident” to 5=“extremely confident” |
| Q8 | Using one word, how would you describe your MAIN response to the fact that you will be exposed to a VR training tool to train stress management skills? | Multiple choice plus open-end response |
| Q9 | Do you believe that a VR training tool will be a helpful and effective training tool in the context of training stress management skills? | Y/N/maybe |
| **M1 (Thoughts Emotions and Behaviours) specific questions** | | |
| Q1 | How much attention and awareness do you pay towards your thoughts and emotions on a daily basis? | Not at all/ some/ a lot |
| Q2 | Are you aware that your thoughts and emotions influence each other and the actions that you take? | Y/N |
| Q3 | How likely are you to actively consider your thoughts and emotions when you respond to a stressful event? | 5-point scale from 1= “not at all likely” to 5= “extremely likely” |
| **M2-5 specific questions** | | |
| Q1 | Are you aware that Controlled Breathing/ PMR/ Grounding/Emotional acceptance is a stress management skill? | Y/N |
| Q2 | Have you ever engaged in Controlled Breathing/ PMR/ Grounding/Emotional acceptance | Y/N |
| Q3 | Please specify the context in which you have engaged in Controlled Breathing/ PMR/ Grounding/Emotional acceptance | Open-end response |
| Q4 | How often have you engaged in Controlled Breathing/ PMR/ Grounding/Emotional acceptance | Multiple choice |
| Q5 | Have you ever applied Controlled Breathing/ PMR/ Grounding/Emotional acceptance specifically prior to, during or after a stressful episode? | Y/N |
| Q6 | How confident are you in engaging with Controlled Breathing/ PMR/ Grounding/Emotional acceptance? | 5-point scale from 1=“not at all confident” to 5=“extremely confident” |
| Q7 | Please specify the context in which you have engaged in Progressive Muscle Relaxation: | Open-end response |
| Q8 | How likely are you to use Controlled Breathing/ PMR/ Grounding/Emotional acceptance when you respond to or after a stressful event? | 5-point scale from 1= “not at all likely” to 5= “extremely likely” |

| **Post-Training Questions for Trainees** | | | |
| --- | --- | --- | --- |
| **Question** | | **Response** | |
| Q1 | Did you complete the VR training module? | Y/N | |
| Q2 | What was the reason for not completing the VR training module? | The VR made me feel sick/ other- open ended questions | |
| Q3 | Were there any technical problems that occurred during the use of the training tool? | Y/N | |
| Q4 | In your opinion, was the information presented during the introduction of this module easy to follow? | Y/N/unsure | |
| Q5 | How well does the introductory section (speech, demeanour of the presenter) align with the ADF training style, environment and values? | 5-point scale from 1= “not at all” to 5= “extremely” | |
| Q6 | How appropriate was the duration of the individual exercised within the module? | To short/ too long/ duration was appropriate | |
| Q7 and 8 | Please rate the level of privacy/ immersion experienced within the VR training module? | 5-point scale from 1= “not at all private/immersive” to 5= “extremely private/immersive” | |
| Q9 | How useful were privacy/ immersion in supporting the training and focus on the training activities? | 5-point scale from 1= “not at all useful” to 5= “extremely useful” | |
| Q9 | In your opinion, what element(s) of the training module have you found most beneficial? | Open-end response | |
| Q10 | In your opinion, what element(s) of the training module could be improved upon? | Open-end response | |
| **M1 (Thoughts, Emotions and Behaviours) specific questions:** | | | |
| Q1 | The VR tool helped me understand how thoughts, emotions and actions are connected. | | 5-point Likert scale 1= “strongly disagree” to 5= “strongly agree” |
| Q2 | Having a clear understanding of my thoughts, emotions and actions is a useful skill for stress reduction | |
| Q3 | This was a useful tool to highlight the importance of being aware of one's thoughts, emotions and behaviours. | |
| Q4 | It wasn't clear what the purpose of the module was. | |
| Q5 | I obtained a deeper understanding of the benefits of being aware of my own thoughts and emotions as a tool to manage stressful situations. | |
| **M2 (Controlled Breathing) specific questions:** | | | |
| Q1 | The VR tool helped me understand changes in my breathing pattern. | | 5-point Likert scale 1= “strongly disagree” to 5= “strongly agree” |
| Q2 | The VR tool provided effective practical training. | |
| Q3 | The VR tool did not make it clear why and when I should engage in controlled breathing. | |
| Q4 | This was a useful tool to develop and practice controlled breathing skills. | |
| Q5 | I am now more competent in controlled breathing. | |
| **M3 (PMR) specific questions:** | | | |
| Q1 | The VR tool helped me understand how to effectively relax parts of my body. | | 5-point Likert scale 1= “strongly disagree” to 5= “strongly agree” |
| Q2 | It was unclear how to connect breathing with muscle tension/ relaxation. | |
| Q3 | The VR tool did not make it clear why and when I should engage in progressive muscle relaxation. | |
| Q4 | The guidance on how to tense and relax each muscle group was clear and easy to follow. | |
| Q5 | I found the module relaxing | |
| Q6 | I am now more competent in progressive muscle relaxation. | |
| Q7 | I feel confident that I can engage in progressive muscle relaxation unguided and outside of the VR headset. | |  |
| **M4 (Grounding) specific questions:** | | | |
| Q1 | The VR tool helped me understand how to regain manual control over my attention and the concept of Grounding | | 5-point Likert scale 1= “strongly disagree” to 5= “strongly agree” |
| Q2 | This was a useful tool to develop and practice Grounding skills | |
| Q3 | The VR tool did not make it clear why and when I should engage in Grounding. | |
| Q4 | I obtained a deeper understanding of the benefits of Grounding as a tool to manage a stressful situation. | |
| Q5 | After using the VR module I am now more competent in Grounding. | |
| Q6 | It was valuable to break up visual, auditory, and sensory grounding into individual exercises. | |
| Q7 | I feel confident that I can engage in Grounding unguided and outside of the VR headset. | |
| **M5 (Emotional acceptance) specific questions:** | | | |
| Q1 | The VR tool did not make it clear how to respond to emotions. | | 5-point Likert scale 1= “strongly disagree” to 5= “strongly agree” |
| Q2 | I obtained a deeper understanding of the benefits of identifying and accepting emotions. | |
| Q3 | I am now more competent in noticing my own emotions. | |
| Q4 | I am now more competent in responding to uncomfortable emotions. | |
| Q5 | I feel confident that I can use non-avoidant strategies unguided and outside of the VR headset. | |
| Q6 | The VR tool provided effective practical training on how to manage emotions. | |
| **Final exposure and overall platform questions:** | | | |
| Q1 | Overall, the VR platform improved my ability to deal with a stressful event. | | 5-point Likert scale 1= “strongly disagree” to 5= “strongly agree” |
| Q2 | Overall, the VR tool provided useful and effective practical training. | | 5-point Likert scale 1= “strongly disagree” to 5= “strongly agree” |
| Q3 | I did NOT improve any practical skills using this module/training. | | 5-point Likert scale 1= “strongly disagree” to 5= “strongly agree” |
| Q4 | How likely are you to engage in Controlled Breathing/ PMR/ Grounding/Emotional acceptance/ consider your thoughts, emotions and behaviours the next time you experience a stressful event? | | 5-point scale from 1= “not at all likely” to 5= “extremely likely” |
| Q5 | When are you likely to use engage in Controlled Breathing/ PMR/ Grounding/Emotional acceptance/ consider your thoughts, emotions and behaviours in the context of a stressful event? | | Before/after/during a stressful event/ all of the above |
| Q6 | Performance Edge is designed to complement the concepts taught in the existing BattleSMART program. Please indicate how well the Grounding module complements the existing program: | | Multiple choice options |
| Q7 | Assuming that Performance Edge will be used for future training with the ADF, when should this content be delivered throughout your career? | | Multiple choice options |
| Q8 | If Performance Edge is available for future training within the ADF, how should the training tool be made accessible to personnel? | | Multiple choice options |

| **Post training questions for training staff** | | |
| --- | --- | --- |
| **Question** | | **Response** |
| Q1 | What is your current Rank/Position? | Open-end response |
| Q3 | How familiar are you with the principles taught in the current BattleSMART program? | 5-point scale from 1= “not at all familiar” to 5= “extremely familiar” |
| Q4 | How aware are you generally of the different strategies and skills that can be used to effectively manage stress? | 5-point scale from 1= “not at all aware” to 5= “extremely aware” |
| Q5 | Do you see value in providing practical stress management skills and strategy training to ADF trainees? | Y/N/Unsure |
| Q6 | Do you see value in repeatedly practicing stress management skills and strategies throughout your life/career? | Y/N/Unsure |
| Q7 | Please rate how confident you are with the general use of virtual reality technology? | 5-point scale from 1= “not at all confident” to 5= “extremely confident” |
| **The following questions required responses based on a 5-point Likert scale ranging from “strongly disagree” to “strongly agree”** | | |
| Q1 | I liked the general design and style of the application. | |
| Q2 | The use of VR technology was interesting and new. | |
| Q3 | I don't believe VR is an appropriate training modality for the ADF. | |
| Q4 | I believe trainees will engage with the VR training. | |
| Q5 | The VR hardware was easy to setup and use. | |
| Q6 | I am confident that I could deliver the VR tool to a classroom. | |
| Q7 | The VR application and the different modules were easy to navigate. | |
| Q8 | I found the VR tool difficult to set up and use. | |
| Q9 | The VR tool delivered valuable knowledge on stress management skills. | |
| Q10 | I don't believe this tool is a suitable method for teaching stress management skills. | |
| Q11 | The purpose of the VR training was unclear. | |
| Q12 | The practical training delivered in VR was  effective. | |
| Q13 | I feel confident that trainees will gain knowledge and develop skills using this tool. | |
| Q14 | The VR tool improved my own knowledge and skills. | |
| Q15 | The VR tool is a waste of time and does not teach skills or knowledge. | |
| Q16 | Performance Edge is a useful addition to BattleSMART. | |
| **Module specific questions:** | | |
| **Q17 How useful was the following module for training stress management skills to ADF personnel:** | | |
| M1 Thoughts, Emotions and Behaviours  M2 Controlled Breathing  M3 Progressive Muscle Relaxation  M4 Grounding  M5 Emotional Acceptance | | 5-point scale from 1= “not at all useful” to 5= “extremely useful” |
| **Question** | | **Response** |
| Q1 | How well did the introductory video (s) (speech, demeanour of the presenter) align with the ADF training style, environment and values? | Multiple choice |
| Q2 | Please rate the level of privacy/immersion experienced within the VR training module? | 5-point scale from 1= “not at all private/immersive” to 5= “extremely private/immersive” |
| Q3 | How useful is privacy/immersion in this particular training context and subject matter? | 5-point scale from 1= “not at all useful” to 5= “extremely useful” |
| Q6 | Do you believe the sense of immersion and privacy contributes to the learning experience within Performance Edge? | Y/N/Maybe |
| **Reflection on overall platform** | | |
| **Question** | | **Response** |
| Q1 | In your opinion, what element(s) of Performance Edge have you found most beneficial? | Open-end response |
| Q2 | In your opinion, what element(s) of Performance Edge could be improved upon? | Open-end response |
| Q3 | The application you saw today is a prototype. Do you see value in further developing this training application? | Y/N/Maybe |
| Q4 | Do you see value in delivering and incorporating Performance Edge into the standard training curriculum? | Y/N/Maybe |
| Q5 | If you selected no or unsure, please provide a brief explanation as to why you see no value in the training delivered in Performance Edge. | Open-end response |
| Q6 | Assuming that Performance Edge will be used for future training with the ADF, when is a suitable time to deliver this training? | Multiple choice (+1 can be chosen) |
| Q7 | If Performance Edge is available for future training within the ADF, how should the training tool be made accessible to personnel? | Multiple choice |
| Q8 | Is there anything you would like to see which would, in your mind, help strengthen the training value of Performance Edge? | Open-end response |
| Q9 | What training area/ subject matter would you like to see included in Performance Edge, as an additional module or included into the existing modules? | Open-end response |
| Q10 | Do you see benefit in capturing performance data within a training group during this training? | Multiple choice |
| Q11 | Would it be useful if the software saved progress, selections and responses for individuals to access outside of the headset? | Multiple choice |
| Q12 | Performance Edge is designed to complement the concepts taught in the existing BattleSMART program. Please indicate how well Performance Edge complements the existing program: | Multiple choice |
